# P046 The tocilizumab dilemma: when guideline-directed therapy isn’t enough in giant cell arteritis

**DOI:** 10.1093/rap/rkag113.076

**Published:** 2026-11-03

**Authors:** Saumya Dinithi Rupasinghe, Roger Bucknall

**Affiliations:** Robert Jones and Agnes Hunt Orthopedic Foundation Trust, Oswestry, United Kingdom; Robert Jones and Agnes Hunt Orthopedic Foundation Trust, Oswestry, United Kingdom

## Abstract

**Introduction:**

Tocilizumab has transformed the management of giant cell arteritis (GCA) by reducing relapse rates and cumulative glucocorticoid exposure. The ideal length of therapy is still up for debate as many patients relapse following treatment withdrawal. In ordinary clinical practice, there is therapeutic ambiguity due to the lack of evidence supporting prolonged or recurrent treatments of IL-6 suppression. We describe a patient with recurrent cranial GCA who repeatedly relapsed following cessation of tocilizumab despite excellent treatment response, underscoring the challenges in striking a balance between disease control, steroid toxicity and current treatment recommendations.

**Case description:**

A 70-year-old white British farm worker presented with periodic transient vision disturbance, jaw claudication, and a worsening left temporal headache. Examination demonstrated left temporal artery tenderness with an ESR of 72 mm/hour, and high-dose prednisolone was commenced immediately for suspected GCA.

Temporal artery ultrasound was normal, although inflammatory markers improved rapidly following glucocorticoid therapy. He had frequent headaches and jaw pain during the corticosteroid taper, which was linked to an increase in inflammatory markers necessitating prednisolone augmentation. Methotrexate was introduced as a steroid-sparing agent.

His treatment course was hampered by steroid-induced hypertension/ impaired blood sugar and subsequent diagnosis of prostate carcinoma requiring radical prostatectomy. The COVID-19 pandemic caused a delay in the planned start of tocilizumab, which eventually commenced in late 2021. His clinical condition significantly improved, enabling a gradual taper of steroids and methotrexate.

Following completion of the recommended treatment course, he experienced a recurrence of temporal headache and jaw claudication, with ESR increasing to 98 mm/hour and positive temporal artery Doppler. Despite repeated relapses, prevailing recommendations did not support treatment beyond one year. However, additional funding applications following multiple discussions enabled a further short course of tocilizumab due to repeated relapses during corticosteroid taper.

PET-CT demonstrated modest uptake without signs of active vasculitis and subsequent temporal artery ultrasound examinations remained normal. Despite these findings, the patient continued to develop recurrent cranial symptoms whenever tocilizumab was withdrawn.

The central question for multidisciplinary discussion was whether repeated or prolonged tocilizumab therapy should be supported despite limited evidence beyond current recommendations.

**Discussion:**

This case demonstrates several important clinical challenges encountered in the management of relapsing GCA. Normal temporal artery ultrasound after corticosteroid exposure does not rule out disease recurrence, even though imaging methods have increased diagnostic precision. Similarly, isolated cranial symptoms may not be correlated with PET-CT results.

Throughout his disease course, clinical relapse consistently followed withdrawal of tocilizumab and improved following reintroduction, suggesting ongoing IL-6-mediated disease activity despite equivocal imaging. This illustrates the drawbacks of relying on individual investigations when managing established GCA.

The patient was exposed to extended high-dose glucocorticoid medication with related side effects, such as hypertension, due to repeated relapses. Despite the proper introduction of methotrexate, repeated disease activity was not prevented. Therefore, the main therapeutic conundrum included striking a balance between the uncertainties surrounding long-term biological therapy and cumulative steroid toxicity.

Current recommendations have historically limited treatment duration because of the absence of robust long-term evidence rather than evidence demonstrating harm. Real-world experience suggests that selected patients with recurrent disease may benefit from longer treatment courses, although the optimal duration remains debatable.

This case demonstrates the importance of individualised decision-making based on previous treatment response, disease severity and patient preference rather than rigid adherence to treatment duration alone. In order to obtain ongoing financing and create a management plan, cooperation between local rheumatology services and specialised centres is crucial.

The key highlight is that the treatment duration for GCA should be customised based on relapse risk rather than being limited to predetermined treatment plans.

**Key learning points:**

1. Relapses following discontinuation of tocilizumab remains a significant challenge in GCA and should be carefully re-evaluated instead of automatic recommencement of steroids.

2. Clinical assessment remains crucial as normal temporal artery ultrasound and equivocal PET findings do not exclude recurrent cranial disease, particularly following steroid exposure.

3. Alternative long-term therapy approaches should be taken into consideration because repeated glucocorticoid escalation carries significant morbidity.

4. Current recommendations regarding treatment duration are based on limited evidence, highlighting the need for individualised management in patients with recurrent disease.

5. Clinicians should promptly balance patient-specific characteristics, prior therapy response, cumulative treatment toxicity, and evidence-based guidelines in real-world practice.

6. When considering long-term biologic therapy outside of conventional therapeutic paths, multidisciplinary cooperation and expert centre input are crucial.

